# Digital educational technology content production and validity about Japanese bathtub (*ofurô*) in neonatal unit

**DOI:** 10.1590/1980-220X-REEUSP-2022-0307en

**Published:** 2023-04-28

**Authors:** Aline Libório de Oliveira, Yasmin Epifânio de Souza, Lihsieh Marrero, Alessandra Pinheiro Vidal, Ana Lua Marinho Freire, Elizabeth Teixeira, Roberta Costa

**Affiliations:** 1 Universidade do Estado do Amazonas, Escola Superior de Ciências da Saúde, Manaus, AM, Brazil.; 2 Universidade Federal do Pará, Belém, PA, Brazil.; 3 Universidade Federal de Santa Catarina, Departamento de Enfermagem, Florianópolis, SC, Brazil.

**Keywords:** Educational Technolog, Infan, Prematur, Bath, Humanization of Assistanc, Nurse Practitioner, Methods, Tecnología Educacional, Recién Nacido Prematuro, Baños, Humanización de la Atención, Enfermeras Practicantes, Métodos, Tecnologia Educacional, Recém-Nascido Prematuro, Banhos, Humanização da Assistência, Profissionais de Enfermagem, Métodos

## Abstract

**Objective:**

To describe the elaboration and content validity stages of a digital educational technology for nursing professionals about Japanese bathtub (*ofurô*) in newborns in neonatal care units.

**Method:**

A methodological study conducted between August 2019 and July 2021, in a public maternity hospital in Manaus, Amazonas, developed in two stages. In the first, the e-book images, text and editing were produced. In the second, material content validity was carried out, through the opinion of 15 expert judges, nurses and neonatal care experts. In data analysis, the percentage of agreement was applied with agreement score estimation. Items with 80% or more agreement were considered valid.

**Results:**

The e-book “Japanese bathtub: manual for nursing professionals” was produced, organized into nine sessions, which describe the technique execution. Content was considered valid by expert judges (general score 90%).

**Conclusion:**

The e-book was considered suitable for use in training nursing professionals, with potential for dissemination of knowledge regarding humanization of care for premature babies.

## RESUMO

**Objetivo:**

Descrever as etapas de elaboração e validação de conteúdo de uma tecnologia educacional digital para profissionais de enfermagem sobre o banho de ofurô em recém-nascidos em Unidades de Cuidado Neonatal.

**Método:**

Estudo metodológico conduzido entre agosto de 2019 e julho de 2021, em uma maternidade pública de Manaus, Amazonas, desenvolvido em duas etapas. Na primeira, foram produzidas as imagens, texto e editoração do *e-book*. Na segunda, foi conduzida a validação de conteúdo do material, pela manifestação de 15 juízes especialistas, enfermeiros e experientes no cuidado neonatal. Na análise dos dados, aplicou-se a porcentagem de concordância com estimação de escore de concordância. Foram considerados válidos os itens com 80% ou mais de concordância.

**Resultados:**

Foi produzido o *e-book* “Banho de ofurô: manual para profissionais de enfermagem”, organizado em nove sessões, que descrevem a execução da técnica. O conteúdo foi considerado válido pelos juízes especialistas (escore geral 90%).

**Conclusão:**

O *e-book* foi considerado adequado para ser utilizado em treinamento de profissionais de enfermagem, com potencial para a difusão do conhecimento quanto à humanização da assistência ao recém-nascido prematuro.

## RESUMEN

**Objetivo:**

Describir las etapas de elaboración y validación de contenido de una tecnología educativa digital para profesionales de enfermería sobre el baño ofurô en recién nacidos en Unidades de Cuidados Neonatales.

**Método:**

Estudio metodológico realizado entre agosto de 2019 y julio de 2021, en una maternidad pública de Manaus, Amazonas, desarrollado en dos etapas. En la primera se produjeron las imágenes, el texto y la edición del e-book. En el segundo, se realizó la validación de contenido del material, a través de la expresión de 15 jueces expertos, enfermeros y experimentados en cuidados neonatales. En el análisis de datos se aplicó el porcentaje de concordancia con estimación del puntaje de concordancia. Se consideraron válidos los ítems con un 80% o más de acuerdo.

**Resultados:**

Se elaboró el libro electrónico “Banho de ofurô: manual para profissionais de enfermagem” (Baño de Ofurô: manual para profesionales de enfermería), organizado en nueve sesiones, que describen la ejecución de la técnica. El contenido fue considerado válido por los jueces expertos (puntuación general 90%).

**Conclusión:**

El e-book se consideró apto para ser utilizado en la formación de profesionales de enfermería, con potencial para la difusión de conocimientos sobre la humanización del cuidado del recién nacido prematuro.

## INTRODUCTION

Annually, 15 million premature births occur in the world, with Brazil among the ten countries with the highest rates^(1)^. Extreme prematurity and very low birth weight, 60.2% and 50.6%, respectively, represent more than half of the causes of neonatal deaths in Brazil^(2)^. As a signatory of the Sustainable Development Goals (SDGs), the country has intensified efforts to reduce neonatal mortality, expanding the number of beds in neonatal units, qualifying professionals and implementing humanized care routines^(3)^.

Births that occur before the 32^nd^ week of gestation are considered extremely premature, a life condition of concern due to its complexity and impact on child growth and development^(2,4)^. Prematurity, when added to very low birth weight (<1,500 grams), poses great challenges to the survival of newborns (NB)^(5)^ who need qualified and humanized assistance. Nursing care for preterm (PTNB) and low birth weight NBs requires mastery of physiological aspects and changes in each phase of child development, skills and ability for safe and humanized care^(6)^.

To minimize the damage caused by prematurity, low weight and hospitalization, neonatal care services seek alternatives in complementary therapies to help PTNB overcome the initial difficulties of life, such as Japanese bathtub (BT), indicated for those with alteration in the neurobehavioral development, in the motor tonus and with difficulty in the organization of the behavioral states^(7)^. This technique consists of immersing the baby in a tub of its own shape containing heated water for 10 minutes, in a vertical and flexed position, simulating the environment of a mother’s womb, providing safety and well-being to NBs, helping in their sensory organization^(8)^.

Despite the recognition of the satisfactory results of BT on PTNBs’ well-being by health professionals and government entities, robust scientific evidence proving the benefits of BT is still scarce. However, some studies already demonstrate that the simulation of an intrauterine environment, promoted by BT has favorable repercussions on the neurobehavioral organization, relief of stress and pain, and improvement of PTNBs’ homeostasis^(6,9,10,11,12)^. In institutional contexts, the technique must be performed by trained professionals, ensuring quality of care and patient safety^(7)^.

Professional training for implementing techniques and practices in health is foreseen in the Brazilian National Permanent Education in Health Policy (*Política Nacional de Educação Permanente em Saúde*), with a view to improving the assistance provided, and must be articulated by the management of institutions, municipal and state health departments, with technical support from the federal sphere of government^(13)^.

Currently, educational forms are growing that make a wide variety of instruments available to health system professionals and users. Smart technologies, together with the standardization of care practices, can contribute to pediatric patient safety. Digital educational technologies (DET) allow easy and quick access to content by the public, given its adaptability to various digital platforms through equipment such as smartphones and tablets, which have fast access to the internet, in addition to having objective and short-term reading duration^(14)^.

DET use on the BT technique as a tool for training nursing professionals who work in Neonatal Care Units (NICU) expands the scope of complementary therapies offered to PTNB, raising the quality of care provided, contributing to neonatal mortality reduction. In this perspective, this study aimed to describe the stages of elaboration and validity of a digital educational technology for nursing professionals about BT in NBs in neonatal care units.

## METHODS

### Study Design

This is a methodological study of DET development and content validity by expert judges. DET describes the BT technique in PTNB, with the target audience being nursing professionals who work in the NICU of a public maternity hospital in Manaus, a reference for the care of high-risk NBs, from August 2019 to July 2021. The study was developed in two stages: DET production and validity of its content.

The first stage was DET production, of e-book type, based on the Standard Operating Procedure (SOP), already available at the institution, designed to guide the execution of BT technique. This stage was conducted between August 2019 and July 2020. The choice of an e-book type DET considered the ease of access and the low cost of producing the material. To compose the e-book, step-by-step illustrations of the technique described in SOP were created in a realistic simulation laboratory, transformed into drawings, followed by theoretical-explanatory text. The first version of the e-book was obtained after editing the material.

The second stage was content validity of the first version of an e-book entitled “Japanese bathtub: manual for nursing professionals”, based on expert judges’ opinion, selected on the *Plataforma Lattes* (https://buscatextual.cnpq.br/buscatextual/busca.do?metodo=apresentar). Using the search filters “academic training/degree”, “professional performance”, profiles that met the inclusion criteria were identified: having a degree in nursing; having a record of academic title; working in the child and adolescent health sub-area. Exclusion criteria were: having resumes in which there was no record of professional, academic and/or scientific experience in the neonatal nursing specialty and/or with BT execution in NB; those for which it was not possible to identify the contact email or that it was returned. A total of 55 potential participants were identified, regardless of the macro-region of the country and state where they operate, to whom an invitation was sent by e-mail announcing the study objectives and research procedures. Of the total number of guests, 22 agreed to participate, with the final sample comprising 15 participants. This stage took place between August 2020 and July 2021, with the purpose of identifying the representativeness of the e-book component items regarding the extension and dimension of the phenomenon of interest that it proves^(15)^. For this reason, because it is a procedure in the health area and because it is a DET aimed at nursing professionals, only professional nurses were elected as expert judges. Only one round of validity was performed.

## DATA COLLECTION

From the SOP, a script was prepared for image production followed by the technique and the explanatory content in text format. This script was validated by two nurses, experts in neonatal nursing, and with experience in performing BT in PTNB, members of the study team.

After adjustments and a second round of script assessment, image production began, with the sequential technique capture statically, using the materials necessary for the procedure and a dummy, simulating the baby’s positions. The images produced were stored, constituting an image bank. The preliminary version obtained needed to be validated before being used in training.

In the second stage, the e-book content was validated based on expert judges’ opinion. An email with an e-book in PDF file, the link to access the Informed Consent Form and the instrument via Google Forms was sent by email to the 22 judges who agreed to participate in this stage. At the end of 30 days, 15 participants completed the form, totaling 15 e-book assessments.

The instrument used for validity was a Likert-type scale^(16)^ with 20 items, organized into three dimensions, “Content”, “Structure and Presentation” and “Relevance”, which allowed assessing the e-book content regarding usefulness, clarity, objectivity, simplicity, up-to-date, vocabulary, and instructional sequence. In the “Content” dimension, DET was assessed by six items regarding the coherence of the information presented, encouraging humanized care, contributing to quality of care, encouraging changes in behavior, meeting the target audience’s needs, and the technique step-by-step description. The “Structure and Presentation” dimension allowed DET validity through the judgment of 13 items, regarding illustration format, adequacy, objectivity, language, logical sequence and expressiveness, textual quality, writing style, information coherence, size and font used, and the number of pages as well as their suitability for the target audience. The “Relevance” dimension consisted of five items for DET validity regarding articulation with health policies, transfer and construction of knowledge, addressing issues necessary for practice and usefulness for training nursing professionals.

The items of each dimension were judged by judges as “Totally Adequate” (TA), “Partially Adequate” (PA) or “Inadequate” (I).

### Data Analysis and Treatment

In the first stage, intended for the e-book production, three researchers with experience in neonatal nursing and in BT execution assessed the images from the database produced and selected the most appropriate ones to compose the e-book. The selected images were transformed into drawings by a designer. The technologies applied to transform the selected images into drawings involved the Photoshop^®^ image editing software, as it offers extensive animation and interaction resources, providing a smaller file size compared to others.

The technique theoretical-explanatory content was extracted from the SOP, organized and inserted into Microsoft Office Word^®^, describing the technique execution coherently with the corresponding image.

In data analysis of DET validity stage, the percentage of agreement method was applied to estimate the agreement score between judges’ responses. Items that obtained a score of 80% or more of agreement were considered valid. The responses’ agreement score ranges from –1 to +1. In this study, the applied score considered as “Consensus” (+1) when 70% or more of the evaluators judged the item as “Totally Adequate” (TA); “Undecided” (0), when 70% or more chose “Partially Adequate” (PA) when assessing the item; and “Dissensus” (–1), when 70% or more rated the item as “Inadequate” (I)^(17)^. When judging the item as PA or I, judges made suggestions for improvement. Data were organized, systematized and analyzed in Excel.

### Ethical Aspects

This study is part of a larger study, approved by the Research Ethics Committee of the *Universidade do Estado do Amazonas* on July 16, 2019 (Opinion 3,456,197), in accordance with Resolution 466 of 2012 of the Brazilian National Health Council and the Brazilian National Commission for Ethics in Research. All participants signed the Informed Consent Form.

## RESULTS

The technique theoretical-explanatory content was organized in an e-book format, given the ease and practicality of access to the content by the public and because it is adaptable technology to different digital platforms, in addition to being an objective material with short-term reading. Material content was assessed by 15 expert judges.

**Figure 1.**
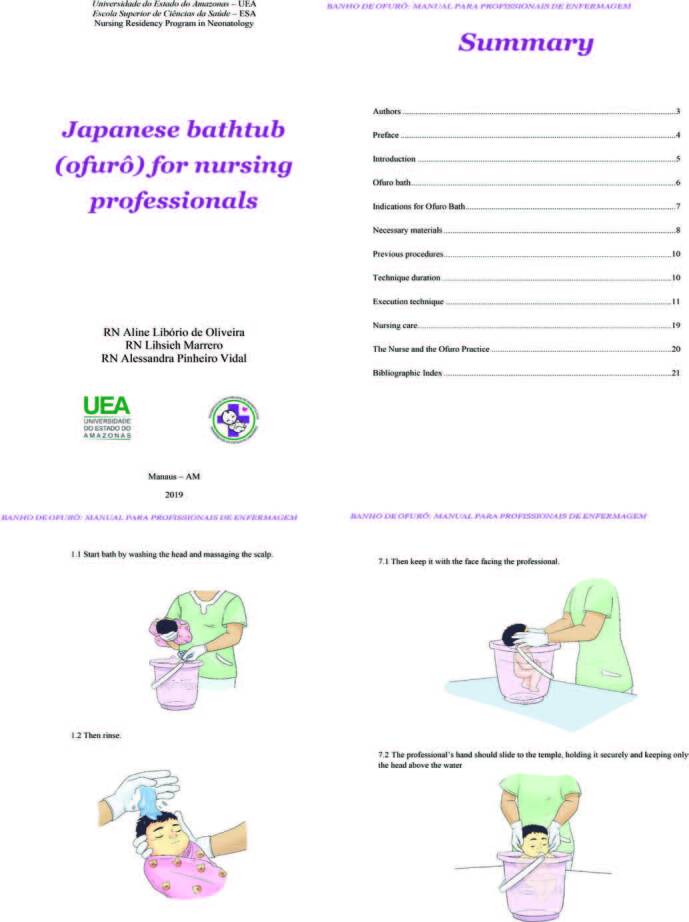
Illustrations from the book “Japanese bathtub: manual for nursing professionals”. Manaus-AM. Brazil, 2021.

The produced DET is illustrated with images followed by textual information, which describe the technique execution. The book “Japanese bathtub: manual for nursing professionals” is organized into nine sections: (1) introduction on the impact of the BT technique procedure for PTNBs; (2) BT concept and its origins; (3) indications for BT in PTNB; (4) materials needed to perform the technique; (5) procedures prior to performing the technique; (6) duration of recommended BT; (7) step by step technique; (8) nursing care; and (9) legal guidelines regarding the technique execution by nursing professionals (Figure 1).

Fifteen judges, aged between 23 and 61 years, female (100%) participated in book content validity. All participants were graduates in nursing. Eight (53.33%) had a doctoral degree, two (13.3%), a master’s degree and five (33.3%), specialization. Regarding the field of action, 53.3% (8) of judges were clinical nurses in neonatal units and 46.7% (7) were nursing professors.

The “Content” dimension of DET was judged on six items regarding the coherence of the information presented, encouraging humanized care, contributing to quality of care, encouraging changes in behavior, meeting the target audience’s needs and describing the steps of the technique step by step. As for the degree of valuation of this dimension, all items obtained “Consensus” (+1) among judges (Table 1).

**Table 1.** Judgment of expert judges of the book “Japanese bathtub: manual for nursing professionals” according to assessment items of “Content”, “Structure and Presentation” and “Relevance” dimensions - Manaus-AM, Brazil, 2021 (N = 15).

	Adequate	Partially adequate	Total	Agreement score
Item	n (%)	n (%)	n (%)	
**Content Dimension**				
Scientific consistency	14 (93.3)	1 (6.7)	15 (100)	+1
Encourages humanized care	13 (86.7)	2 (13.3)	15 (100)	+1
Contributes to care	13 (86.7)	2 (13.3)	15 (100)	+1
Encourages behavior changes	14 (93.3)	1 (6.7)	15 (100)	+1
Meets the target audience’s needs	15 (100)	–	15 (100)	+1
Describes the technique step-by-step	14 (93.3)	1 (6.7)	15 (100)	+1
**Structure and Presentation Dimension**				
Meets the audience’s needs	15 (100)	–	15 (100)	+1
Objectivity of conveyed message	13 (86.7)	2 (13.3)	15 (100)	+1
Language used	13 (86.7)	2 (13.3)	15 (100)	+1
Logical sequence of illustrations	14 (93.3)	1 (6.7)	15 (100)	+1
Textual quality	12 (80)	3 (20)	15 (100)	+1
Writing style used	12 (80)	3 (20)	15 (100)	+1
Size and font used in the text	11 (73.3)	4 (26.7)	15 (100)	+1
Illustration expressiveness	13 (86.7)	1 (6.7)	15 (100)	+1
Number of pages	14 (93.3)	2 (13.3)	15 (100)	+1
**Relevance Dimension**				
Articulation with health policies and guidelines	13 (86.7)	2 (13.3)	15 (100)	+1
Knowledge transfer	13 (86.7)	2 (13.3)	15 (100)	+1
Knowledge building	14 (93.3)	1 (6.7)	15 (100)	+1
Addresses practical issues	12 (80)	3 (20)	15 (100)	+1
Usefulness for training	13 (86.7)	2 (13.3)	15 (100)	+1

The “Structure and Presentation” dimension consisted of nine items referring to illustration format, adequacy, objectivity, language, logical sequence and expressiveness, textual quality, writing style, consistency of information, size and fonts used, number of pages in the digital book and coherence with the target audience. In this dimension, DET obtained the “Consensus” (+1) of judges’ opinions (Table 1).

The “Relevance” dimension was assessed in five items: articulation with health policies, transfer and construction of knowledge, approach to issues for practice and usefulness for training nurses. The items obtained the score of “Consensus” (+1) among judges’ responses (Table 1).

In the assessment of the whole, the DET obtained an overall score of 90%, being considered valid and able to be used in training of nursing professionals who work in neonatal units with PTNB. Of the 20 items in the validity instrument, none item was assessed as “Inadequate”, with no need for another assessment round.

**Chart 1.**
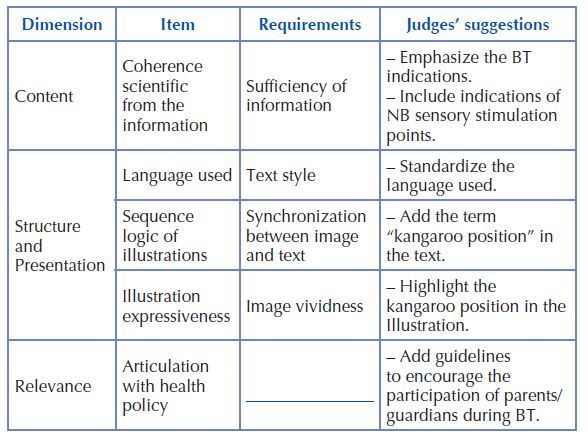
Expert judges’ suggestions for changes according to items in the digital book “Japanese bathtub: manual for nursing professionals” – Manaus, Amazon, Brazil, 2021 (N = 15).

In judges’ opinion, the “Content” dimension in the “scientific coherence of information” item, specifically regarding the “sufficiency of information” requirement, needed changes. The suggestions for changes received were related to the inclusion of information regarding bath time and points for sensory stimulation of NB during the procedure (Chart 1).

In the “Structure and Presentation” dimension, the suggestions for alteration, related to the “language used” item, were the requirement “textual style”, which presented a need for standardization. The item “logical sequence of illustrations” also received suggestions for improvement in the “synchrony between images and texts” requirement in one of the figures. In the “illustration expressiveness” item, the “image vividness” requirement received a suggestion to change the image to highlight the kangaroo position (Chart 1).

The “Relevance” dimension received as a suggestion for alteration in the “articulation with the health policy” item, the inclusion of guidelines to professionals to encourage the participation of parents/guardians during the procedure (Chart 1).

Expert judges’ suggestions were incorporated into the second version of DET. A limitation of this study is the need for DET to be validated in terms of appearance and usability, including judges from other areas and the target audience.

## DISCUSSION

Educational technology production is a tool for the process of improving nursing care. In the age of communication, the internet is a simple and fast means of accessing diverse content. In this context, DET are presented as a differentiated form of teaching-learning^(18)^. In this context of educational innovations to subsidize humanized care practices for NBs, the development of a TED, describing the BT technique in PTNB, in participating expert judges’ opinion, proved to be valid to be used in neonatal nursing care qualification.

The neonatal unit environment has an impact on PTNBs’ development due to excessive lights, noise and manipulation, causing discomfort and pain, expressed by physiological and behavioral changes. In this regard, humanized techniques and practices in the hospital environment that NB care in recovery provide neurocognitive and sensory protection for NBs, minimizing the development of deficiencies common to prematurity, favoring healthy development and reducing hospitalization time^(10,19)^. Nurses’ qualification to perform the BT technique contributes to expanding the offer of humanized care in neonatal units, in line with national and international guidelines for neonatal mortality reduction.

A literature review study that analyzed the contributions of DET use in teaching nursing skills, observed that DET are increasingly present in the teaching of the area, with a wide variety of technological innovations that collaborate in the development of clinical skills in nursing, such as digital didactic materials^(20,21)^. Bearing in mind that learning does not end with the completion of graduation, these technologies also incorporate knowledge into the development of professional practices.

From this perspective, the results of this study present a DET validated by expert judges, being suitable for use in training nurses in the technique execution. Educational material validity by experts in the area guarantees the scientific content coherence, attributing credibility to the product^(16)^. The DET developed in this study had its content-related items considered adequate by expert judges, as it articulates images and texts, offering access to the information necessary for understanding the message and assimilating the conveyed information.

The “Relevance” dimension consensus assessment highlights the potential of the digital book as an instrument that facilitates the knowledge and development of the appropriate technique for Japanese bathtub bath in PTNBs, addressing its main points for professional qualification, by providing access to reliable and safe information and knowledge about the technique. Educational tools in health, such as the DET developed in this study, combined with active methodologies and the internet, complete innovative teaching-learning strategies by incorporating portability, mobility and connectivity accessible to users^(22,23)^.

Furthermore, the technologies and instruments available for health interventions must be designed in order to give voice to the target audience, providing valid information, which was reinforced in the study considering nursing professionals’ scientific and empirical knowledge^(24)^. The strategy of using technology in neonatal care is directed towards respecting and valuing human life, rethinking all forms of the team’s relationship with the baby and their family, promoting adequacy of knowledge, personified, safe and ethical care^(25)^.

The study main limitation is that DET was not applied to the target audience, being a stage in development by the larger research team. However, DET validity on the BT technique is a step forward for the area of nursing and health, expanding the possibilities for incorporating complementary therapies in PTNB care.

## CONCLUSIONS

Articulating the need to have an educational technology and the technological evolution, the book “Japanese bathtub: manual for nursing professionals” was produced, validated by expert judges, being available for training programs for these professionals to perform the BT technique in neonatal units.

Japanese bathtub is a shared practice among the different professions in the health area, not being exclusive to any category, which is gaining ground within neonatal care units, but still requires greater concentration of research to be included in nursing practices as a procedure with scientifically proven benefits.

The developed material will support the qualification process of nursing professionals for the technique execution and implementation in neonatal units, expanding humanized care to preterm NBs, offering well-being, contributing to reducing length of stay in the institution and neonatal mortality.
